# Functionalized Gold Nanoparticles with a Cohesion Enhancer for Robust Flexible Electrodes

**DOI:** 10.1021/acsanm.2c00742

**Published:** 2022-04-25

**Authors:** Jisun Im, Gustavo F. Trindade, Tien Thuy Quach, Ali Sohaib, Feiran Wang, Jonathan Austin, Lyudmila Turyanska, Clive J. Roberts, Ricky Wildman, Richard Hague, Christopher Tuck

**Affiliations:** † Centre for Additive Manufacturing, Faculty of Engineering, University of Nottingham, Jubilee Campus, Nottingham NG8 1BB, U.K.; ‡ Advanced Materials and Healthcare Technologies, School of Pharmacy, University of Nottingham, University Park, Nottingham NG7 2RD, U.K.

**Keywords:** gold nanoparticles, conductive ink, cohesion, inkjet printing, flexible electronics, additive manufacturing

## Abstract

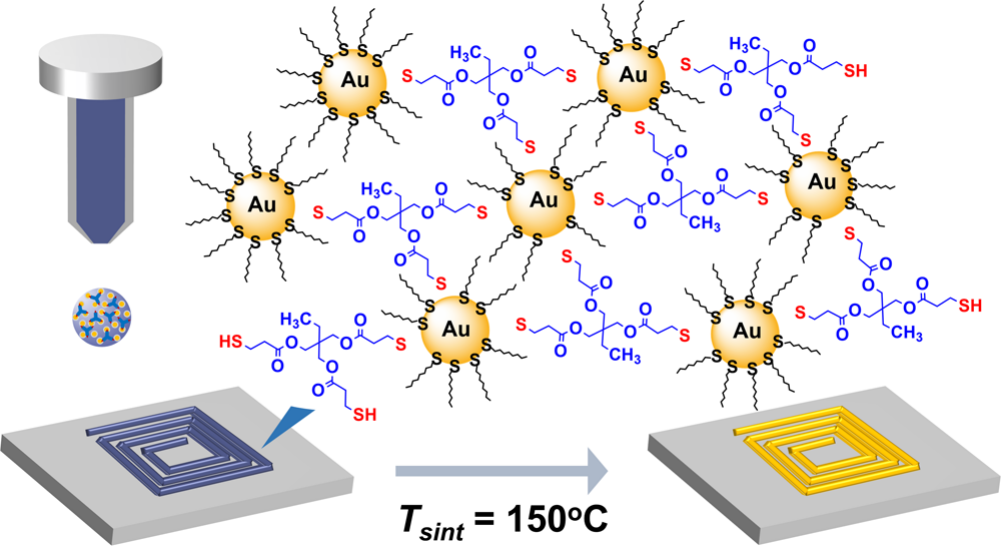

The development of conductive inks is required to enable additive manufacturing of electronic components and devices. A gold nanoparticle (AuNP) ink is of particular interest due to its high electrical conductivity, chemical stability, and biocompatibility. However, a printed AuNP film suffers from thermally induced microcracks and pores that lead to the poor integrity of a printed electronic component and electrical failure under external mechanical deformation, hence limiting its application for flexible electronics. Here, we employ a multifunctional thiol as a cohesion enhancer in the AuNP ink to prevent the formation of microcracks and pores by mediating the cohesion of AuNPs via strong interaction between the thiol groups and the gold surface. The inkjet-printed AuNP electrode exhibits an electrical conductivity of 3.0 × 10^6^ S/m and stable electrical properties under repeated cycles (>1000) of mechanical deformation even for a single printed layer and in a salt-rich phosphate-buffered saline solution, offering exciting potential for applications in flexible and 3D electronics as well as in bioelectronics and healthcare devices.

## Introduction

There is a strong demand for conductive inks to enable the additive manufacturing of a new generation of functional electronics, including printed and flexible electronics, wearable and healthcare electronics, and consumer electronics.^1−3^ Various types of conductive inks have been developed, including those containing metals (e.g., silver,^4,5^ gold,^6−8^ copper,^9,10^ and so forth), carbon allotropes (e.g., graphene,^11,12^ CNT,^13,14^ and so forth), and conductive polymers (e.g., PEDOT:PSS).^15,16^ Of particular interest are conductive inks based on metal nanoparticles (NPs), which can be sintered at a lower temperature compared to that of the corresponding bulk metal due to the high surface area to volume ratio,^17^ and they exhibit relatively high electrical conductivities.^18^

To date, research efforts have mostly focused on silver NP inks. However, highly mobile Ag ions generated in the presence of heat and applied electric fields are capable of diffusion through pinholes in the SiO_2_ layer, which has detrimental effect on the quality of the gate in Si/SiO_2_-based electronic devices.^19,20^ A gold NP (AuNP) conductive ink has the potential to overcome these limitations. Also, the chemical stability and biocompatibility of gold are particularly advantageous for bioelectronics that are operational in harsh environments, such as high humidity and salt-rich fluids (e.g., body sweat).^21^

Despite the body of work, the NP-based layer suffers from thermally induced microcracks and pores, causing device failure.^22,23^ The attempts to resolve this issue focused on pressure- or ultrasonic-assisted sintering.^24,25^ However, there is still a strong demand for new conductive inks, which will enable the deposition of stable and durable layers.

In this work, we develop a AuNP ink formulation with a cohesion enhancer, which prevents the formation of microcracks and pores and enables the deposition of stable layers with respect to their morphological and electrical properties. AuNP functionalization with a cohesion enhancer can be produced by a two-step method: (i) synthesis of octanethiol-functionalized AuNP (OT-AuNPs) and (ii) ligand exchange reaction. The Brust method is used for the synthesis of OT-AuNPs since it reliably produces large quantities of relatively monodisperse NPs.^26,27^ The OT-AuNPs are further functionalized with a multifunctional thiol, trimethylolpropane tri(3-mercaptopropionate) (TrisSH), via ligand exchange. The AuNP layers deposited with TrisSH produce more uniform and continuous printed structures due to improved cohesion of AuNPs. The role of TrisSH acting as a cohesion enhancer is confirmed by advanced compositional analysis using Orbitrap secondary ion mass spectrometry (OrbiSIMS) and X-ray photoelectron spectroscopy (XPS). This AuNP ink is compatible with silicon-based electronics, and deposition of the AuNP ink on the Si/SiO_2_ substrates does not affect the gate, unlike with silver NP inks. We demonstrate the benefits of enhanced stability of this AuNP ink for applications requiring flexible substrates, where we achieve stable conductivity over 1000 bending cycles, as well as operational stability in salt-rich aqueous environments. This work proposes a novel strategy for the formulation of stable and flexible metal NP inks with enhanced cohesion and could advance their exploitation in wearable and healthcare electronics.

## Results and Discussion

### Gold NP Ink Formulation and Inkjet Printing

**Figure 1:**
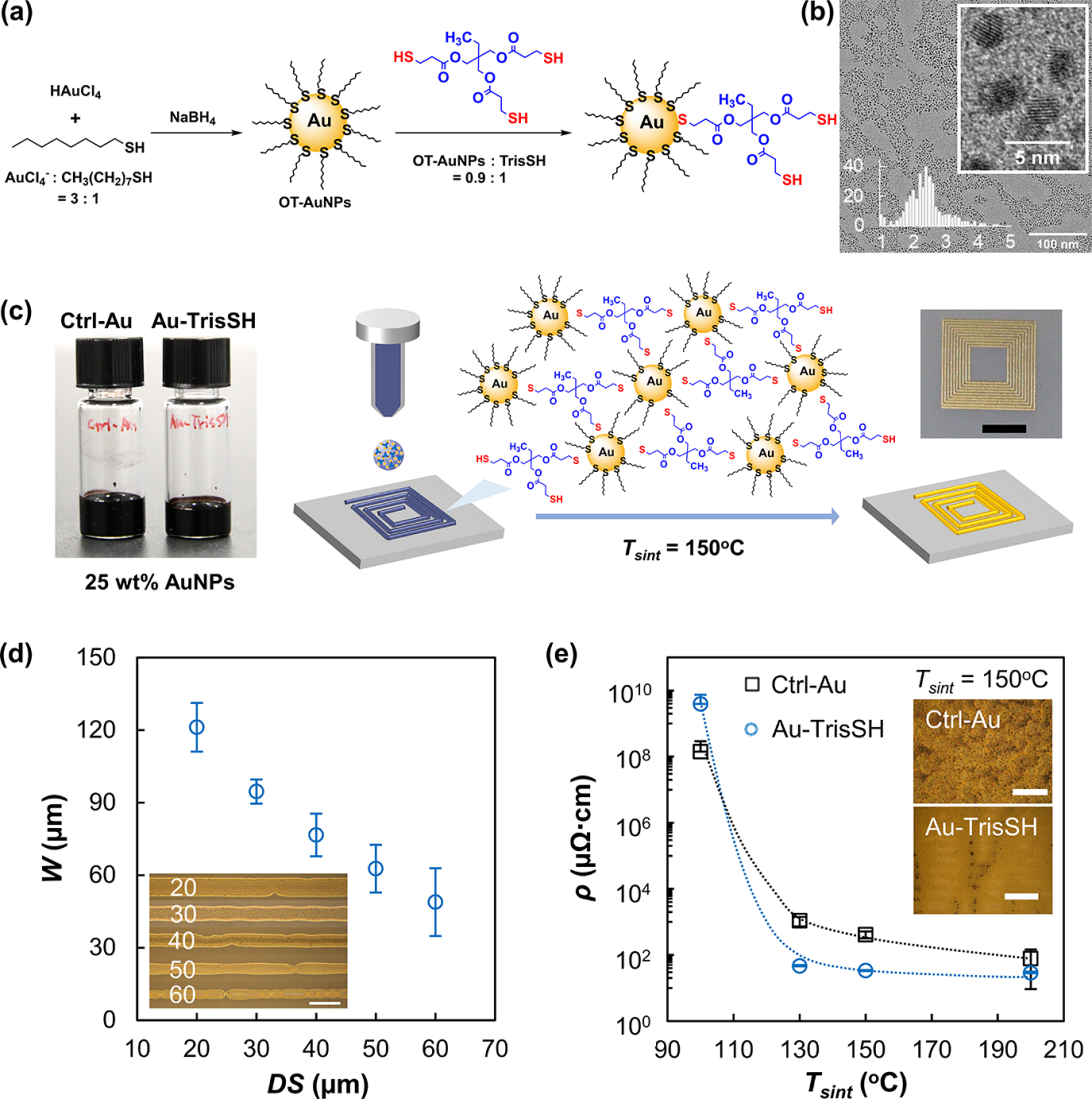
(a) Schematic of the synthesis of OT-AuNPs and the ink formulation with a multifunctional thiol (TrisSH) as a cohesion enhancer. (b) Representative TEM and (top inset) HRTEM images of OT-AuNPs. (lower inset) Histogram of the size distribution of OT-AuNPs. (c) (left) Photograph of two ink formulations, Au-TrisSH and Ctrl-Au, with and without TrisSH. (right) Schematic of inkjet deposition of the Au-TrisSH ink and (inset) a photograph of an inkjet-printed gold square-planar spiral coil (scale bar is 3 mm). (d) Dependence of the line widths (*W*) of the Au-TrisSH ink deposited on Si/SiO_2_ substrates on DS values. (inset) Optical microscopy image of AuNP lines printed at different DS values (the scale bar is 200 μm). (e) Dependence of electrical resistivity (ρ) of printed Ctrl-Au and Au-TrisSH lines on sintering temperatures (*T*_sint_) for 30 min. The data points represent the mean and standard deviation for at least three independent measurements. (inset) Optical microscopy images of Ctrl-Au and Au-TrisSH lines post-treated at *T*_sint_ = 150 °C (the scale bar is 200 μm). The dotted lines are a guide to the eye.

The OT-AuNPs were synthesized by the modified Brust method (Figure 1a).^26^ The average core diameter of OT-AuNPs in the range of 1–3 nm was obtained at a 3:1 AuCl_4_^–^/octanethiol molar ratio, which was confirmed from transmission electron microscopy (TEM) images (Figure 1b). XPS revealed characteristic Au 4f_7/2_ for Au(0) and S 2p_3/2_ for gold-thiolate bonding at 84 and 162 eV, respectively (Figure S1), confirming the successful synthesis of the OT-AuNPs. Thermogravimetric analysis (TGA) of the OT-AuNPs showed that the desorption and volatilization of octanethiolates from the surface of AuNPs began at 200 °C and was completed at 280 °C in air (Figure S2). As observed from TGA, the average mass fraction of octanethiolates in the AuNPs was calculated to be 17.6 wt %. The ratio of octanethiolates to surface Au atoms was calculated to be 0.61 by the model detailed by Murray et al.^28^ using the average particle diameter and the mass fraction of octanethiolates in the AuNPs.

To achieve enhanced cohesion between the NPs and to prevent the formation of defects during sintering, we introduce a multifunctional thiol, TrisSH, to the AuNP ink. We propose that TrisSH can act as a cohesion enhancer, where thiol groups can bind to the surface of neighboring AuNPs via a ligand exchange reaction with octanethiolates. We formulated two AuNP inks, Au-TrisSH and Ctrl-Au, by dispersing 25 wt % (1.6 vol %) of OT-AuNPs in terpineol (mixture of isomers) with and without 0.125 wt % of TrisSH, respectively. The dispersion of OT-AuNPs in the Au-TrisSH ink with a 0.9:1 AuNPs/TrisSH molar ratio (0.125 wt % of TrisSH) was stable for more than 8 months at room temperature without agglomeration and precipitation of AuNPs in the dispersion due to the presence of TrisSH (Figure S3). We envisage that the binding of the terminal SH groups of TrisSH to neighboring NPs becomes possible when the neighboring NPs are in close vicinity due to solvent evaporation after being deposited on a substrate (Figure 1c).

The inkjet printing resolution of the AuNP inks was studied on the following substrates: a borosilicate glass slide, silicon wafer (Si/SiO_2_), poly(ethylene terephthalate) (PET), and poly(ethylene naphthalate) (PEN). The average single droplet diameters (one pixel size) of Ctrl-Au and Au-TrisSH inks deposited on Si/SiO_2_ at room temperature were measured to be 78 ± 1 and 62 ± 1 μm, respectively (Figure S4). A smaller droplet size (67 ± 3 μm) was achieved on all substrates investigated at the substrate temperature of 90 °C due to rapid solvent evaporation and pinning on substrates. Continuous printed lines were deposited with the drop spacing (DS) values ranging from 20 to 40 μm (Figure 1d). A larger DS of 50 μm led to the formation of irregular bulging, while a DS of 30 μm enabled the deposition of lines with a continuous and uniform surface, as observed using an optical microscope, with an average line width of 95 ± 5 μm. Hence, a DS of 30 μm and a substrate temperature of 90 °C were chosen to produce samples for further studies. Focused ion-beam scanning electron microscopy (FIB-SEM) images of the cross section of a single-layer printed gold structure revealed a layer thickness of 163 ± 24 nm (Figure S5).

### Electrical Properties of Inkjet-Printed Gold Structures

The sintering temperature (*T*_sint_) affects the electrical properties of NP-based inks. The electrical resistivity of a single-layer sample (10 mm × 0.5 mm) printed on Si/SiO_2_ and sintered at varying temperatures was measured in a four-point geometry, which allows the reduction of the contact resistance. Contacts were produced using silver paint (see the Experimental Section). A significant decrease of electrical resistivity (ρ) by up to 6 and 8 orders of magnitude was observed for both Ctrl-Au and Au-TrisSH samples, respectively, after sintering at *T*_sint_ = 130 °C (for 30 min) compared to *T*_sint_ = 100 °C (Figure 1e). We note that at *T*_sint_ = 150 °C, suitable for flexible polymer substrates, ρ = 33.5 ± 1.4 μΩ cm was observed for a single printed layer of Au-TrisSH, which is over 1 order of magnitude lower than that for Ctrl-Au (ρ = 414.3 ± 96.8 μΩ cm). The achieved electrical resistivity for a single-layer Au-TrisSH is comparable to the reported values for commercial gold inks: (i) ρ ∼ 20 μΩ cm at *T*_sint_ = 200 °C^7^ and (ii) ρ ∼ 22.7 μΩ cm at *T*_sint_ = 130 °C in combination with photonic sintering.^8^

The activation energy (0.14 kJ/mol) of the sintering process for Au-TrisSH is lower than 0.85 kJ/mol for Ctrl-Au in the *T*_sint_ range from 130 to 200 °C (Figure S6), which results in the lower resistivity of Au-TrisSH than that of Ctrl-Au. The activation energies of Au-TrisSH and Ctrl-Au in the *T*_sint_ range from 100 to 130 °C were calculated to be 11.0 and 7.1 kJ/mol (similar to the reported value of 9.1 kJ/mol for octanethiol ligands on AuNPs with an average diameter of 2.2 nm),^29^ respectively, which are higher than those in the *T*_sint_ range from 130 to 200 °C because of more octanethiolates remaining on the deposited film at a lower *T*_sint_. The higher activation energy of 11.0 kJ/mol for Au-TrisSH compared to that for Ctrl-Au (7.1 kJ/mol) in the *T*_sint_ range from 100 to 130 °C is likely due to the longer chain length of TrisSH incorporated on the gold surface via ligand exchange with octanethiolates. The electrical properties of these printed layers remain stable over a period of at least 1 year (sheet resistance change < 4%). We note that our AuNP inks can be used on Si/SiO_2_ substrates without any negative effects of the gate, which are typical for silver.^30^ Indeed, in the inkjet-printed Au/SiO_2_/Si structure, the gate is preserved up to ±100 V of the applied gate voltage under ambient conditions, proving that no migration and diffusion of Au occur through the SiO_2_ layer of a 200 ± 20 nm thickness (Figure S7).

**Figure 2:**
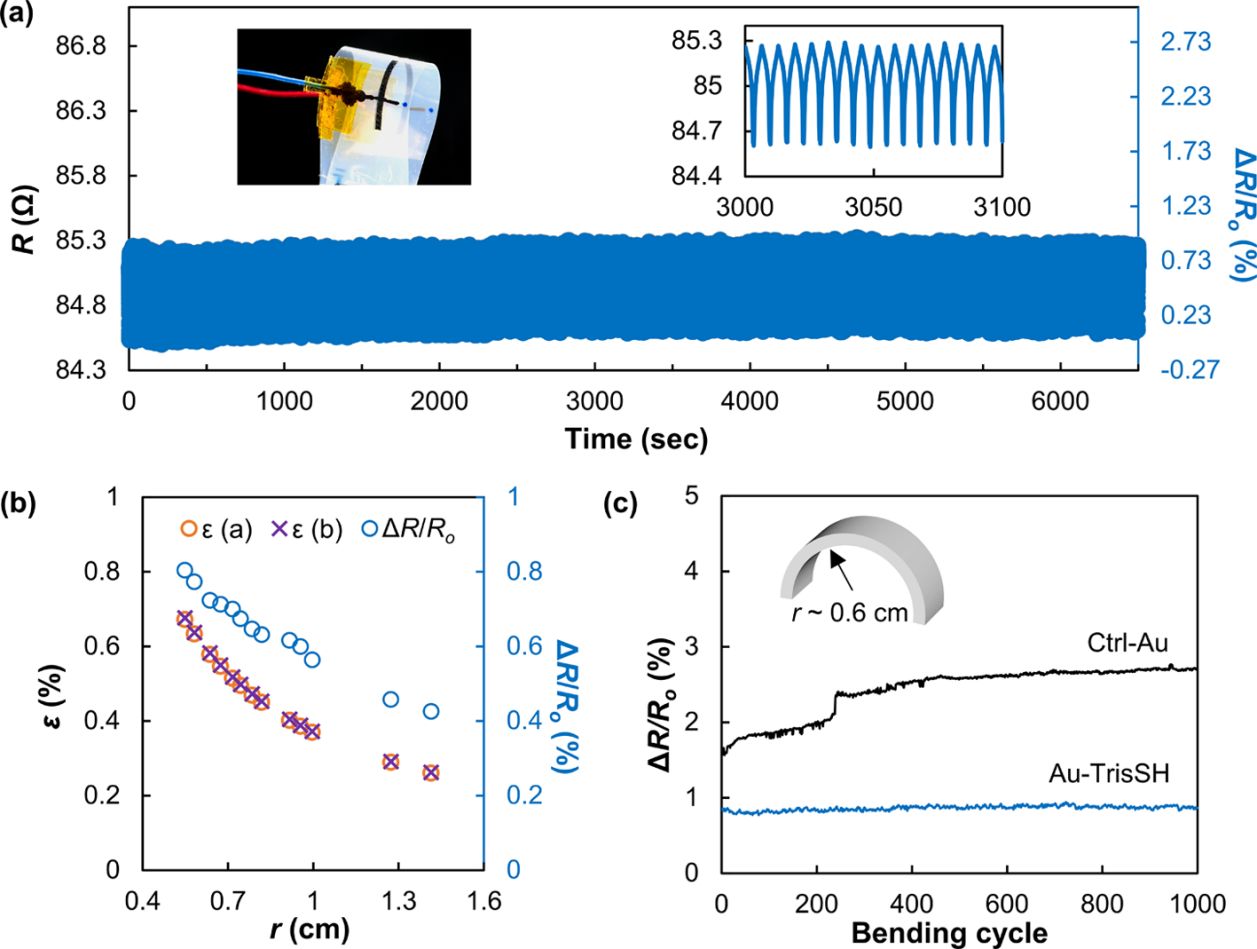
Electrical performance stability study of single printed layers of Ctrl-Au and Au-TrisSH on a PEN substrate (*T*_sint_ = 150 °C). [(a) and right inset] Electrical resistance (*R*) and normalized electrical resistance change (Δ*R*/*R*_o_) measured over 1000 bending cycles for the Au-TrisSH electrode. (left inset) Photograph of an inkjet-printed Au-TrisSH electrode on PEN. (b) Dependence of the calculated bending strain (ε) and Δ*R*/*R*_o_ on the bending curvature (*r*) for the Au-TrisSH electrode. (c) Representative Δ*R*/*R*_o_ dependence of Ctrl-Au and Au-TrisSH electrodes on the number of bending cycles (*r* = 0.6 cm).

For flexible electronics applications, it is critical that the conductive layers maintain electrical properties when subjected to mechanical deformation. Cyclic bending deformation studies were performed on single-printed-layer electrodes (20 mm × 1 mm) on a PEN substrate sintered at 150 °C. The electrical resistance (*R*) was monitored through 1000 cycles of bending to the curvature *r* ≤ 0.6 cm. A stable electrical performance (Δ*R* = 0.03 Ω) was observed for the Au-TrisSH electrode over 1000 cycles of bending (Figure 2a) compared to that for the Ctrl-Au electrode (Δ*R* = 3 Ω) (Figure S8). The bending strain (ε) in the convex surface was calculated from eq 1. ε(*a*) and ε(*b*) were calculated from Young’s modulus values of bulk Au and thin Au film, respectively. The Δ*R*/*R*_o_ values of the Au-TrisSH electrode examined at *r* = 0.6 cm (ε = 0.7%) and *r* = 1.4 cm (ε = 0.3%) were found to be 0.8% and 0.4%, respectively (Figure 2b). The Au-TrisSH electrode exhibited 1.85% lower Δ*R*/*R*_o_ compared to that of the Ctrl-Au electrode (Figure 2c), with the Ctrl-Au-printed electrode showing an abrupt increase in electrical resistance in the ∼240th cycle of bending. This can be ascribed to mechanical degradation induced by tensile strain on the printed Au electrode and stress concentration at microcracks and pores formed within the printed microstructures. The higher bending strain tolerance of the Au-TrisSH electrode compared to that of Ctrl-Au is most likely due to the enhanced cohesion and more continuous and denser microstructure of the printed electrode by the incorporation of a cohesion enhancer.

The good adhesion between an inkjet-printed thin metal film and a flexible substrate is also essential for device integrity and reliable performance. Surface pretreatment of substrates such as silanization or thiol modification prior to inkjet printing of AuNPs has been reported to improve the adhesion.^6,31^ We report here that the adequate adhesion of an inkjet-printed Au-TrisSH thin film to a flexible polymer substrate can be achieved by adding organic solvents with similar solubility parameters (such as Hansen) to the polymer into the ink formulation. Organic solvents such as toluene and xylene can partially dissolve the polymer substrates (PET and PEN) and create an intermixing layer between Au and substrates, which results in good adhesion between a printed AuNP film and a substrate. An inkjet-printed Au-TrisSH film (a single layer) from the AuNP dispersion [25 wt % OT-AuNPs and 0.125 wt % TrisSH in a xylene/terpineol mixture (3:7 w/w)] passed the Scotch tape peel test with sheet resistance (*R*_s_ ∼ 6.5 Ω/sq after peel test), increasing by ∼8 times compared to *R*_s_ ∼ 0.8 Ω/sq before the peel test (Figure S9). Addition of xylene into the ink formulation does not affect the stability and jettability of the Au-TrisSH dispersion.

**Figure 3:**
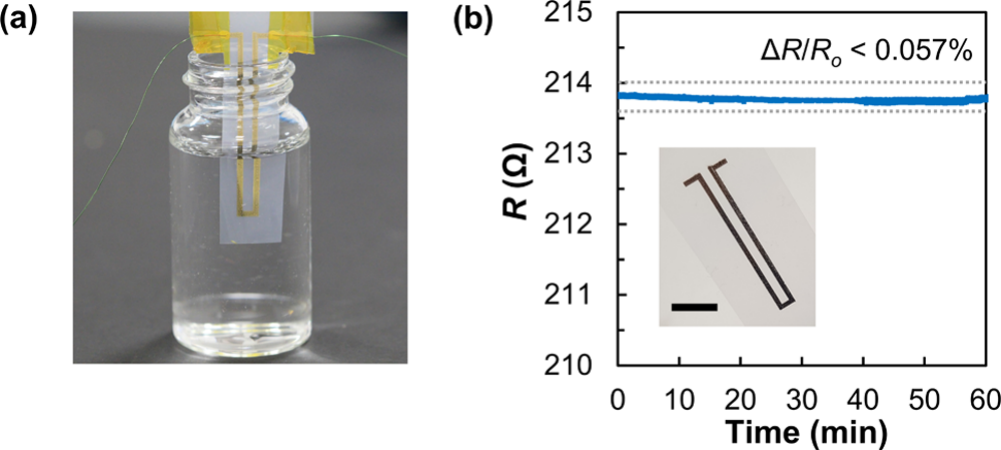
(a) Photograph of a single-layer printed Au-TrisSH electrode on a PEN substrate and a test setup in the PBS solution and (b) its electrical resistance (*R*) monitored for 1 h with Δ*R*/*R*_o_ (%) < 0.057%. (inset) Photograph of the inkjet-printed Au-TrisSH electrode (the scale bar is 1 cm).

The Au-TrisSH ink also offers opportunities for applications requiring chemical stability. We utilized a printed Au-TrisSH electrode in a salt-rich phosphate-buffered saline (PBS) solution (sodium chloride 137 mM, potassium chloride 3 mM, disodium hydrogen phosphate 8 mM, and potassium dihydrogen phosphate 1.5 mM) and found that electrical resistance was maintained for 1 h (Figure 3) with only a marginal decrease (<0.2 Ω) of the resistance, corresponding to Δ*R*/*R*_o_ ∼ 0.057%. This high-electrical-performance stability of Au-TrisSH in a PBS solution (commonly used in biological research) indicates its applicability to bioelectronic application.

### Effect of a Cohesion Enhancer on a Printed Microstructure

**Figure 4:**
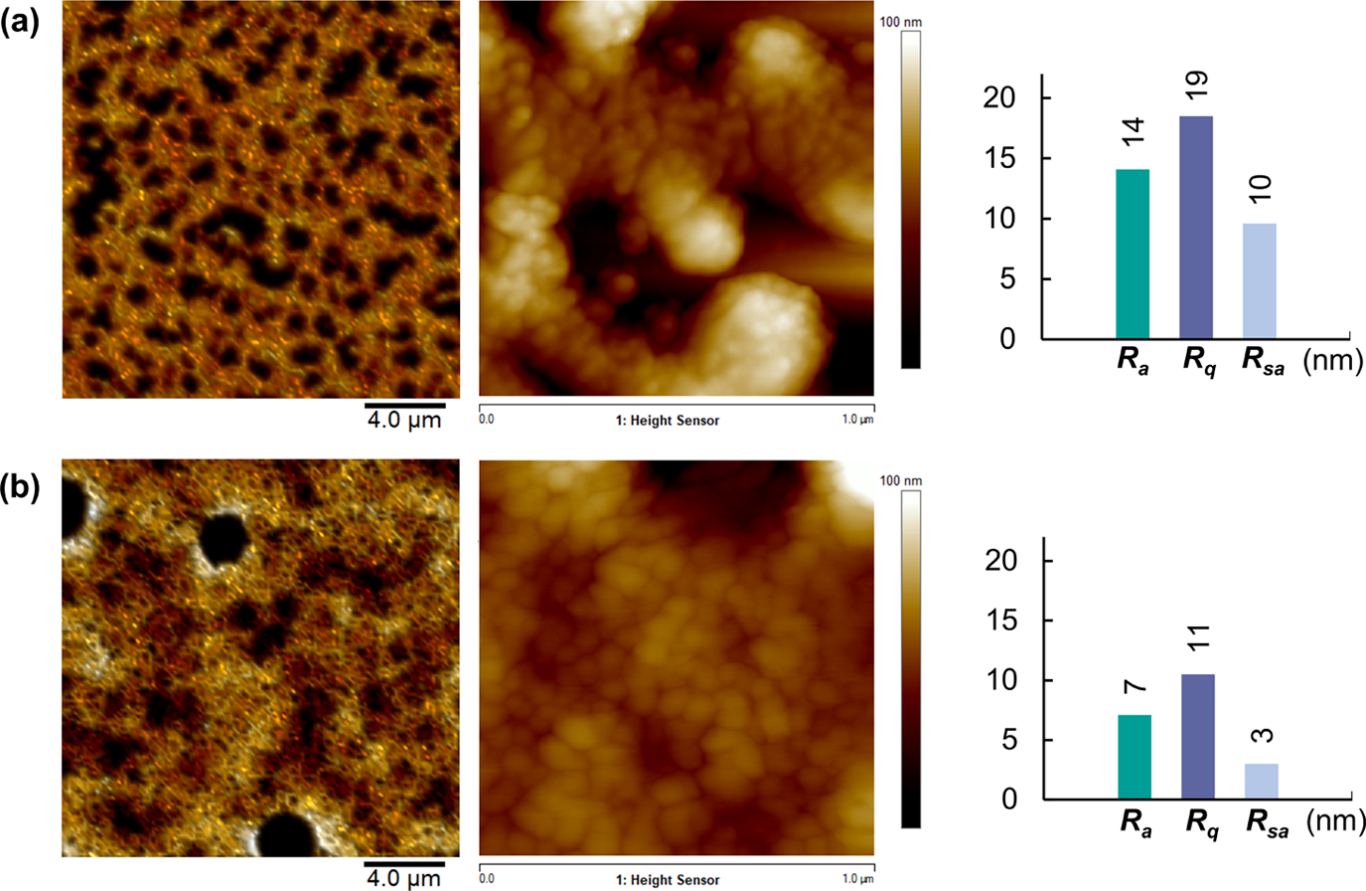
Representative AFM images of the surface morphology and microstructure characterization of single-layer printed gold structures (Si/SiO_2_ substrate, *T*_sint_ = 150 °C) of (a) Ctrl-Au and (b) Au-TrisSH. The left images were acquired using the PeakForce tapping mode (20 μm × 20 μm); the middle images show the HR images over 1 μm × 1 μm (tapping mode); the right graphs show the surface roughness values, *R*_a_, *R*_q_, and *R*_sa_, estimated from 1 μm × 1 μm images.

To elucidate the role of a cohesion enhancer in improved electrical and mechanical properties, we investigated the chemical composition and presence of microstructures in the inkjet-printed Au-TrisSH compared to that in the Ctrl-Au. Atomic force microscopy (AFM) of the Ctrl-Au (single layer on the Si/SiO_2_ substrate, *T*_sint_ = 150 °C) (Figure 4a) revealed a porous structure with pore diameters in the range of 1–2 μm and the average surface roughness *R*_a_ = 14.1, root-mean-squared surface roughness *R*_q_ = 18.5, and surface area increase roughness *R*_sa_ = 9.6 (estimated from 1 μm × 1 μm AFM images). The formation of the pores is driven by the coalescence of NPs at high *T*_sint_ to reduce their surface energy and decrease the surface area during sintering.^32^ In contrast, the surface of the Au-TrisSH layer was found to be more continuous and denser compared to that of Ctrl-Au with reduced roughness values of *R*_a_ = 7.1, *R*_q_ = 10.5, and *R*_sa_ = 3.0 (Figure 4b). These results indicate that TrisSH helps to produce a more uniform and smooth printed gold layer. A considerable difference in the grain microstructures of the printed layers was also noted, where nonuniform large grains were observed in the Ctrl-Au layer compared to those in Au-TrisSH. The more uniform smaller grains observed for Au-TrisSH can be attributed to the role of TrisSH bound to neighboring NPs, which results in denser NP packing and enhanced cohesion. AuNPs without TrisSH are likely to have increased mobility on the substrate during sintering, leading to the formation of larger grains and pores within the printed structure. We note the universality of our ink formulation, whereby the roughness of the Au layers is comparable when deposited on a polished Si/SiO_2_ substrate (surface roughness typically <1 nm) and on a PEN film. The thickness of the deposited Au film is measured to be 163 ± 24 nm (Figure S5) and is thicker than the surface roughness of typically used surfaces (such as polymers and glass), so the nature of a substrate is not expected to considerably affect the surface roughness of the deposited layer.^33^

**Figure 5:**
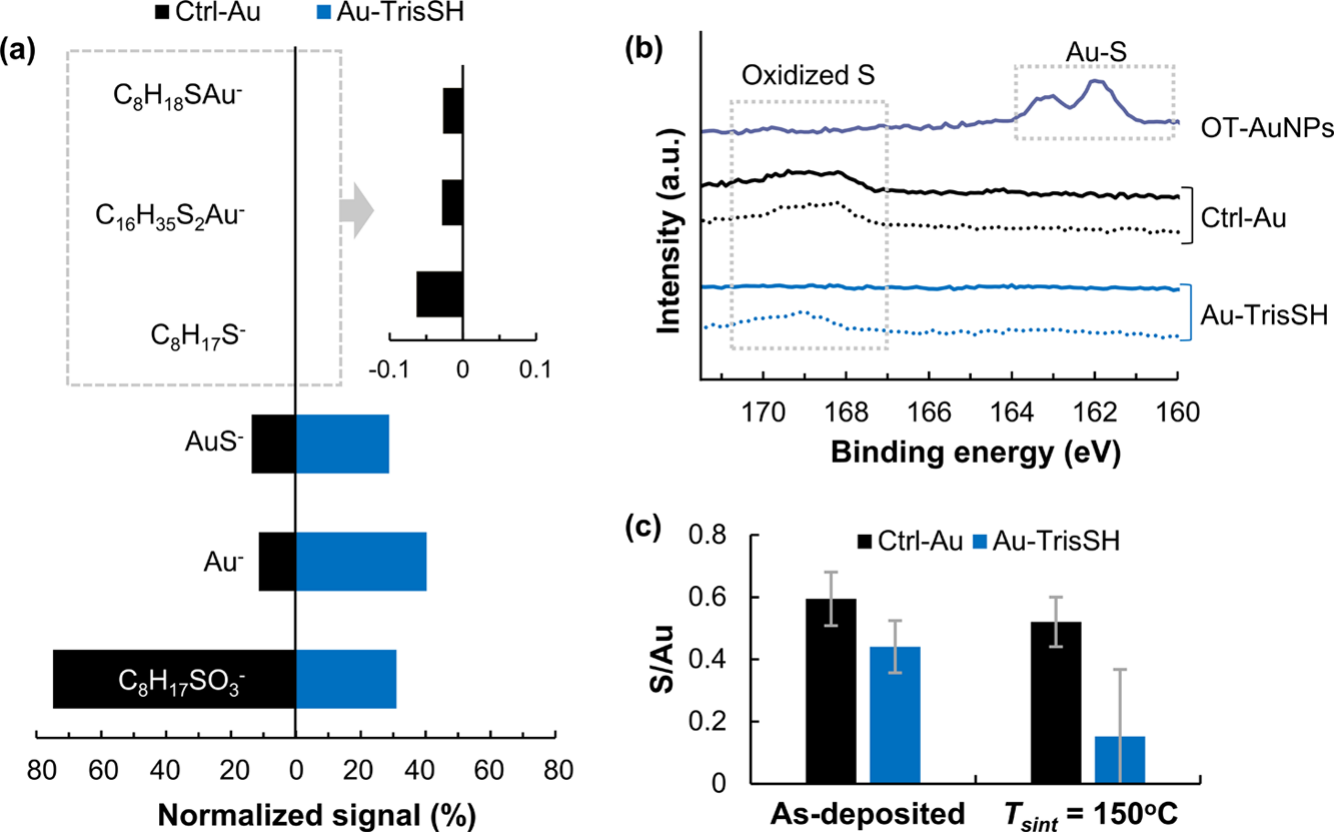
Chemical composition of single-layer printed gold structures of Ctrl-Au and Au-TrisSH (Si/SiO_2_ substrate, *T*_sint_ = 150 °C). (a) Normalized OrbiSIMS data showing Au, octanethiolates, Au-octanethiolate clusters, and oxidized ligand species from the surfaces of the printed structures. (b) XPS S 2p core-level spectra of the Ctrl-Au- and Au-TrisSH-printed samples as-deposited at 90 °C (dotted lines) and sintered at 150 °C (solid lines) compared with those of OT-AuNPs. (c) S/Au atomic ratio of the Ctrl-Au- and Au-TrisSH-printed samples.

To confirm the role of TrisSH as a cohesion enhancer, the surface chemical and molecular composition of printed structures was studied using OrbiSIMS^34^ and XPS. From the OrbiSIMS data, very weak signals were detected for octanethiolate molecular ions (C_8_H_17_S^–^) and Au-thiolate clusters (C_8_H_18_SAu^–^ and C_16_H_35_S_2_Au^–^) for the Ctrl-Au sample only, while sulfonate species (alkylsulfonate, C_8_H_17_SO_3_^–^) were observed for both samples (Figures 5a and S10), indicating the cleavage of Au-thiolate bonding and the oxidation of octanethiolates. The absence of octanethiolate molecular ions and Au-thiolate clusters and the weaker signal for the sulfonate species relative to Au in Au-TrisSH are indicative of a higher level of ligand desorption in this sample. The conversion of thiolates to alkylsulfonates lowers their affinity to gold NP surfaces and makes them more susceptible for desorption. Also, grain coarsening could lead to further release of organic ligands,^35^ contributing to the formation of more uniform layers. The findings of OrbiSIMS results are corroborated by XPS high-resolution (HR) S 2p core-level spectra of the printed structures of Ctrl-Au and Au-TrisSH before and after sintering at 150 °C. Figure 5b shows that octanethiolates of the OT-AuNPs were oxidized in both printed samples even at relatively low temperature of 90 °C (as-deposited). After treatment at *T*_sint_ = 150 °C, the oxidized ligands remain almost the same on the surface of Ctrl-Au, whereas the desorption of most sulfonate species and hence reduced quantities of organic residues were observed for the Au-TrisSH-printed sample (Figure 5c).

**Figure 6:**
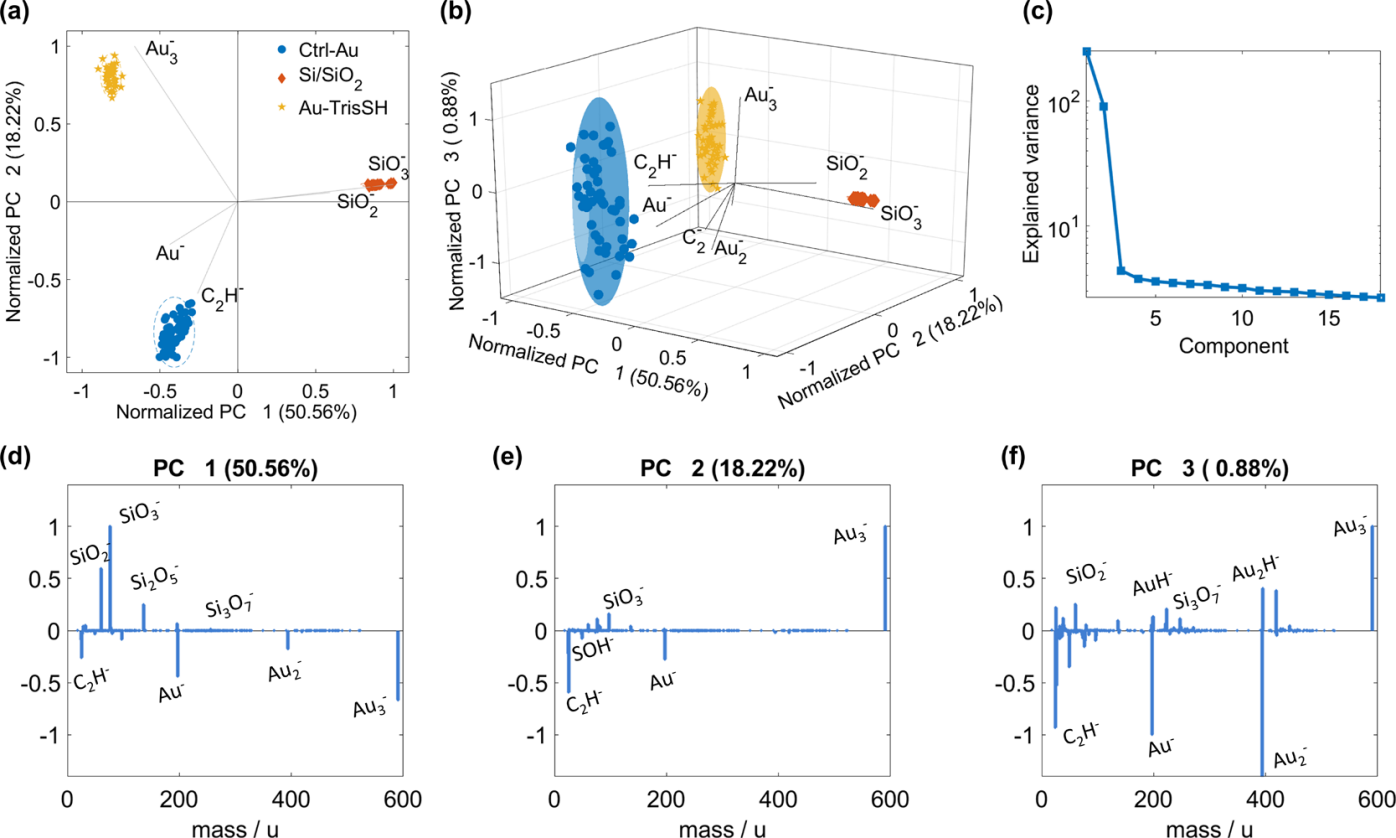
PCA of ToF-SIMS data on the surfaces of the single-layer printed gold structures of Ctrl-Au and Au-TrisSH (Si/SiO_2_ substrate, *T*_sint_ = 150 °C). (a) 2D and (b) 3D score scatter plots of principal components PC1, PC2, and PC3. (c) Explained variance per principal component. Score loadings of (d) PC1, (e) PC2, and (f) PC3.

The improved cohesion and integrity of AuNP owing to TrisSH are also supported by principal component analysis (PCA) of time-of-flight secondary ion mass spectrometry (ToF-SIMS) data (Figure 6). The PCA method seeks to reduce the dimensionality of a dataset containing many variables (mass peaks) down to a few factors (principal components). This allows for the interpretation of the surface chemistry by providing data that can be directly assigned to fingerprint mass spectral peaks of compounds (loadings) and their relative intensities (scores) for each type of samples.^36^ The scatter plots of the scores (Figure 6a,b) and loading plots (Figure 6d,e) show that the first principal component (PC1) separates the signals from Au and a Si/SiO_2_ substrate. Anticorrelation between Au^–^ and Au_3_^–^ observed in the second principal component (PC2) loading indicates the presence of oxygen that increases the ionization probability of Au^–^ in relation to Au_3_^–^.^37^ According to the AFM results in Figure 4, a Si/SiO_2_ substrate is more exposed in the Ctrl-Au sample, which agrees to a higher Au^–^ identified in the PCA results. PC2 also separates Ctrl-Au from Au-TrisSH with the loading plot of PC2 (Figure 6e), revealing that the secondary ions of C_2_H^–^ and SOH^–^ were detected for the Ctrl-Au-printed layer with greater intensity compared to the Au-TrisSH layer, which is consistent with OrbiSIMS and XPS results (Figure 5), showing more organic residual found in Ctrl-Au. The third principal component (PC3) is shown for comparison (Figure 6b,f) and captured very little variance (Figure 6c) without providing any extra information. From this compositional analysis, we conclude that lower electrical resistivity and greater stability of Au-TrisSH compared to those of Ctrl-Au can be attributed to a greater degree of ligand removal during post-treatment and the formation of continuous layers with significantly reduced porosity, as seen in the surface morphology (Figure 4).

## Conclusions

In this study, we have demonstrated a gold conductive ink formulation that can produce robust printed electronic components. We introduced the cohesion enhancer, multifunctional thiol (TrisSH), in the gold ink to prevent the formation of microcracks and pores by mediating the cohesion of AuNPs via a strong interaction between the thiols and the gold surface. We observed lower porosity and lower electrical resistivity for the TrisSH-functionalized AuNP ink. The presence of TrisSH facilitates the formation of more uniform layers with lower porosity during postprocessing. Under thermal sintering conditions, these ligands are more effectively removed from the AuNP layer, resulting in the reduced amount of dielectric organic ligands and enabling NPs to form conductive channels. The ability to produce more uniform and continuous printed structure due to improved cohesion of AuNPs was evidenced by AFM imaging of the surface morphology and surface chemical composition analysis using OrbiSIMS, ToF-SIMS, and XPS. The gold ink with the cohesion enhancer exhibited improved electrical conductivity and high electrical stability under mechanical deformation and in a salt-rich aqueous solution, which makes it a promising conductive material for flexible printed electronics and bioelectronic application.

## Experimental Section

### Materials

All chemicals were purchased from Sigma-Aldrich and used without further purification. PBS tablets (Thermo Scientific Oxoid) were purchased from Thermo Scientific and used for the preparation of a PBS solution (sodium chloride 137 mM, potassium chloride 3 mM, disodium hydrogen phosphate 8 mM, and potassium dihydrogen phosphate 1.5 mM). PEN (75 μm thickness) and PET (125 μm thickness) films were supplied by GTS Flexible Materials Ltd. Silicon wafers single side polished, ⟨100⟩, p-type (boron-doped), and a SiO_2_ layer thickness of 200 nm) were purchased from PI-KEM Ltd.

### Synthesis of Gold NPs

The synthesis of octanethiol-functionalized AuNPs was performed by the modified Brust method.^26^ Gold(III) chloride trihydrate (0.0104 mol, HAuCl_4_·3H_2_O) in the aqueous phase (50 mL) was transferred using a phase-transfer agent, tetraoctylammonium bromide [0.021 mol, (C_8_H_17_)_4_NBr], into the octanethiol (0.0035 mol) solution in toluene (100 mL) using a 3:1 AuCl_4_^–^/thiol molar ratio. Aqueous sodium borohydride (0.104 mol, NaBH_4_) was then added dropwise while stirring, and the mixture was stirred vigorously for 3 h under ambient conditions. The dark, violet-colored organic phase was separated from the aqueous phase, rotary-evaporated to 15 mL, and diluted to 300 mL with ethanol. The product (yield 89%) was washed five times with ethanol and separated by centrifugation and dried under vacuum.

### Ink Formulation

Two gold inks, Au-TrisSH and Ctrl-Au, were formulated with and without trimethylolpropane tri(3-mercaptopropionate) as the cohesion enhancer, respectively. 25 wt % (1.6 vol %) of the OT-AuNPs was dispersed in terpineol (mixture of isomers). 0.125 wt % of TrisSH was added to produce Au-TrisSH ink formulation. The particles were dispersed using a bath sonicator for 30 min and stored in the dark at ambient temperature before use.

### Inkjet Printing

The inks were deposited using a Fujifilm Dimatix Materials Printer (DMP-2850) with a 10 pL cartridge (DMC-11610). A nozzle temperature of 35 °C was used to generate a stable droplet. Different substrate temperatures ranging from 20 to 90 °C were used. For electrical property measurement, the inks were printed using one nozzle, with a DS of 30 μm, a jetting frequency of 1 kHz, a substrate temperature of 90 °C, and a sintering temperature of 150 °C (30 min, hot plate). The sintering temperature dependence of the electrical resistivity of a printed structure was studied by placing a sample on a hot plate at varying temperatures.

### Characterization

TEM studies were performed using JEOL 2100F FEG TEM operating at an accelerating voltage of 200 kV. ImageJ software was used to analyze the particle size. For TGA, PerkinElmer TGA4000 was used, and the samples were heated from 40 to 800 °C at a rate of 20 °C/min in air. The optical microscopy images were acquired using an optical microscope (Nikon Eclipse LV100ND). FIB-SEM imaging was performed to measure the thickness of a printed gold structure using Zeiss Crossbeam XB550. For this study, a sample was coated with a 8 nm-thickness layer of iridium using a coater (Q150R Plus-Rotary Pumped Coater, Quorum) under an argon atmosphere. During SEM imaging, a secondary electron (SESI) detector was operated at 2 kV and 100 pA to acquire cross-sectional images (with SmartSEM software, Zeiss). To differentiate a gold layer in the top view and side view, a gas injection system in Crossbeam 550 was used to deposit a carbon layer onto the targeted position before applying a gallium-FIB milling.

### Electrical Property Measurement

For electrical resistivity measurement, a single-layer sample (10 mm × 0.5 mm) was printed on a Si/SiO_2_ substrate and sintered at varying temperatures (100, 130, 150, and 200 °C) for 30 min using a hot plate. The *I*–*V* characteristics were measured using Keithley 2400/2401 source meters in a four-point geometry, where four contacts were made using conductive silver paint (RS Components); a pair of external contacts was used to apply current, while an inner pair was used for voltage measurement, allowing the exclusion of any effect of contact resistance. We note that measurements using two probe geometry gave comparable results. The electrical resistivity (ρ) was calculated using the following equation: ρ = *R*(*Wt*/*L*), where *R* is the resistance from the *I*–*V* curve, *W* is the width, *t* is the thickness, and *L* is the length of the printed line. *L* and *W* were measured using an optical microscope, and *t* was measured to be 163 ± 24 nm for a single printed layer from a FIB-SEM image of the cross-section of a printed structure.

Single-layer samples of 20 mm (*L*) × 1 mm (*W*) on a PEN substrate sintered at 150 °C with two contacts (Ag paint) at the end were used for electrical performance stability during mechanical bending. The bending setup mainly comprised a stepper motor-based linear stage with 3D printed grips for mounting the sample. The stage (drylin SHTP-01-06) was a lead screw driven with a maximum travel range of 15 mm. The two ends of a sample were mounted between the end block and the moving carriage on the stage, and the speed, length, and number of bending cycles were controlled using a DRV8825 stepper driver. The electrical resistance of a printed electrode was continuously monitored using Keithley 2401 through bending cycles. The normalized electrical resistance change (Δ*R*/*R*_o_) was calculated using the equation Δ*R*/*R*_o_ = (*R* – *R*_o_)/*R*_o_, where *R* is the resistance of a printed electrode after bending and *R*_o_ is that before bending (at the flat state). The bending strain (ε) in the convex surface was calculated using the following equation^38^
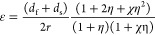
1where subscripts f and s denote the film and substrate, respectively; η = *d*_f_/*d*_s_, where *d*_f_ and *d*_s_ denote the thickness of the film and substrate, respectively; χ = *E*_f_/*E*_s_, where *E*_f_ and *E*_s_ denote Young’s modulus of the film and substrate, respectively. For calculation of the bending strain, Young’s modulus (*E*_f_) values of 77.2, 54, and 7 GPa were used for bulk gold,^39^ thin gold film,^40^ and PEN,^41^ respectively.

### Adhesion Test

The Scotch tape peel test was performed to estimate the adhesion of an inkjet-printed Au film on a polymer substrate. A single layer of a Au-TrisSH film (0.2 mm × 11 mm) was printed on a PEN substrate using the AuNP dispersion including 25 wt % OT-AuNPs and 0.125 wt % TrisSH in a xylene/terpineol mixture (3:7 w/w) and sintered at 150 °C for 30 min. The tape peel test was carried out by placing a 3M Scotch tape (Scotch Magic Invisible tape) and peeling it off. The adhesion was estimated from an optical microscope image and the sheet resistance change measurement after the peel test.

### Surface Analysis

XPS measurements of the AuNP films and printed patterns were performed using an AXIS Ultra Instrument (Kratos) with a monochromated Al Κα X-ray source (1486.6 eV) operated at a 10 mA emission current and 12 kV anode potential (120 W). The spectra were acquired using the Kratos Vision II software. A charge-neutralizer filament was used to prevent surface charging. High resolution spectra at a pass energy of 20 eV, a step of 0.1 eV, and sweep times of 10 min each were also acquired for photoelectron peaks from the detected elements. All spectra were charge-corrected to the C 1s peak (adventitious carbon/CH_2_) set to 284.8 eV. Peak fitting of S 2p scans was performed using the Casa XPS software.

AFM (Dimension Icon, Bruker) with NanoScope Analysis software was used with two modes: (1) PeakForce mapping in air with a scan size of 20 μm, a scan rate of 0.2 Hz (512 lines), a PeakForce setpoint of 3.3 nN, and a PeakForce amplitude of 50 nm and (2) Tapping in air with a scan size of 1 μm, a scan rate of 2 Hz (256 lines), an amplitude set point of 10 mV, and a drive amplitude of 50 mV. The two AFM tips (from Bruker), RTESPA 150 (with a medium force measurement of 6 N/m and a tip radius of 8 nm) and RTESPA 300 (with a high force measurement of 40 N/m and a tip radius of 8 nm), were used for PeakForce and tapping imaging in air, respectively.

Surface ToF-SIMS and OrbiSIMS of the printed gold samples were carried out using a 3D OrbiSIMS (Hybrid SIMS) instrument. The ToF-SIMS spectra were acquired in the negative ion polarity mode using a 20 keV Bi_3_^+^ primary ion beam delivering 0.3 pA. The primary ion beam was raster-scanned over different areas with the total ion dose kept under the static limit of 10^13^ ions/cm^2^. A low-energy (20 eV) electron flood gun was employed to neutralize charge build up. The OrbiSIMS spectra were acquired using a 20 keV Ar_3000_^+^ GCIB of 20 μm diameter and delivering 3.5 nA (with the duty cycle set to 70.4%) was used as the primary ion beam. Argon gas flooding was used to aid charge compensation, and the pressure in the main chamber was maintained at 9.0 × 10^–7^ bar. The spectra were collected in negative polarity in a mass range of 50–750 *m*/*z*. The mass resolving power was 218,726 at 200 *m*/*z*. All ToF-SIMS data were normalized by the total ion counts to correct for topographic features. To perform PCA, mapping data of the surfaces of Ctrl-Au, Au-TrisSH, and a silicon wafer (Si/SiO_2_) substrate were stitched into a single map and arranged in a matrix containing the mass spectral peak areas in columns and pixels in rows using the simsMVA software.^36^ The method seeks to reduce the dimensionality of a dataset down to a few factors, which allows for the interpretation and visualization of the surface chemistry by providing data that can be directly assigned to the fingerprint mass spectra peaks of compounds and their distribution maps. Details of the data preprocessing methodology can be found elsewhere.^42^

## Supporting Information Available

Characterization data of OT-AuNPs including the XPS HR spectra of Au 4f and S 2p core levels, TGA, stability of ink dispersions, printing resolution at different substrates and substrate temperatures, FIB-SEM data of the cross-section of a printed electrode, Arrhenius plots of electrical conductivities of printed structures, leakage current test of the Au/SiO_2_/Si structure, picture of the bending test setup, normalized electrical resistance change of the Ctrl-Au electrode versus time over 1000 bending cycles, adhesion test results, and OrbiSIMS data (mass spectra and peak assignment table) (PDF)
