# Supplementary material for: Functionalized Gold Nanoparticles with a Cohesion Enhancer for Robust Flexible Electrodes
Source: ACS Appl Nano Mater. 2022 Apr 25;5(5):6708–16. doi: 10.1021/acsanm.2c00742 (PMC9150063; doi:10.1021/acsanm.2c00742)
Supplement: Supplementary file 1 — an2c00742_si_001.pdf [file an2c00742_si_001.pdf]

Supporting Information

Functionalized Gold Nanoparticles with Cohesion Enhancer for Robust Flexible Electrodes

Jisun Im, [1]*, *Gustavo F. Trindade,*[1,2] *Tien Thuy Quach,*[1,2] *Ali Sohaib,*[1] *Feiran Wang,*[1] *Jonathan Austin,*[1] *Lyudmila Turyanska,*[1] *Clive J. Roberts,*[2] *Ricky Wildman,*[1]*Richard Hague*[1] *and Christopher Tuck*[1]*

[1]Centre for Additive Manufacturing, Faculty of Engineering, University of Nottingham, Jubilee Campus, Nottingham, NG8 1BB, United Kingdom

[2]Advanced Materials and Healthcare Technologies, School of Pharmacy, University of Nottingham, University Park, Nottingham, NG7 2RD, United Kingdom

Corresponding authors:

Christopher Tuck: Christopher.Tuck@nottingham.ac.uk

Jisun Im: Jisun.Im@nottingham.ac.uk

1. Characterization of octanethiol-functionalized gold nanoparticles (OT-AuNPs)

1.1. X-ray photoelectron spectroscopy (XPS) characterization

XPS analysis on OT-AuNPs was used to evidence Au oxidation states (Au(0)) and the presence of octanethiolate on the Au surface. Au4f and S2p core level spectra are shown in Figure S1. XPS revealed characteristic $Au4f_{7/2}$ for Au(0) and $S2p_{3/2}$ for gold-thiolate bonding at 84 eV and 162 eV, respectively, confirming the successful synthesis of OT-AuNPs.

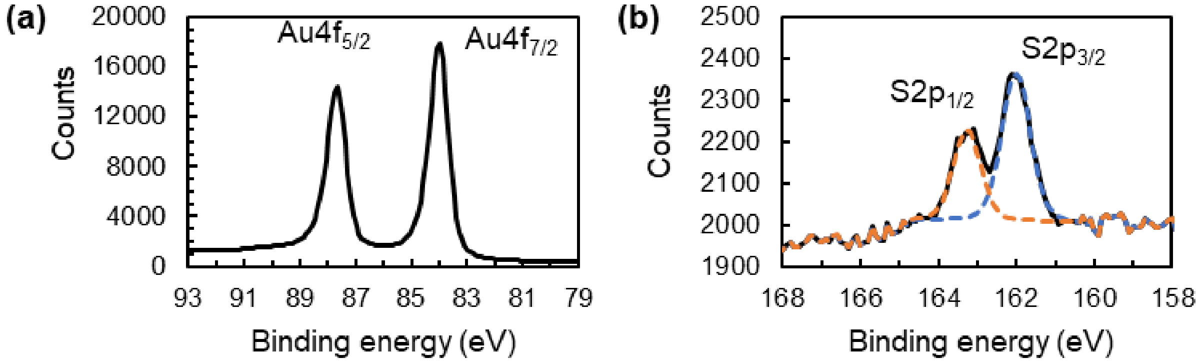

Figure S1. XPS high resolution spectra of (a) Au4f and (b) S2p core levels of OT-AuNPs.

1.2. Thermogravimetric analysis (TGA)

TGA was used to investigate the ligand desorption onset temperature and estimate the ligand weight percentage of OT-AuNPs by heating samples from 40ºC to 800ºC at a rate of 20ºC/min in air. Figure S2 shows that octanethiolates begin desorption from the surface of gold at 200ºC and all ligands are completely desorbed and decomposed at 280ºC in air. The average mass fraction of octanethiolates in AuNP was calculated to be 17.6 wt%.

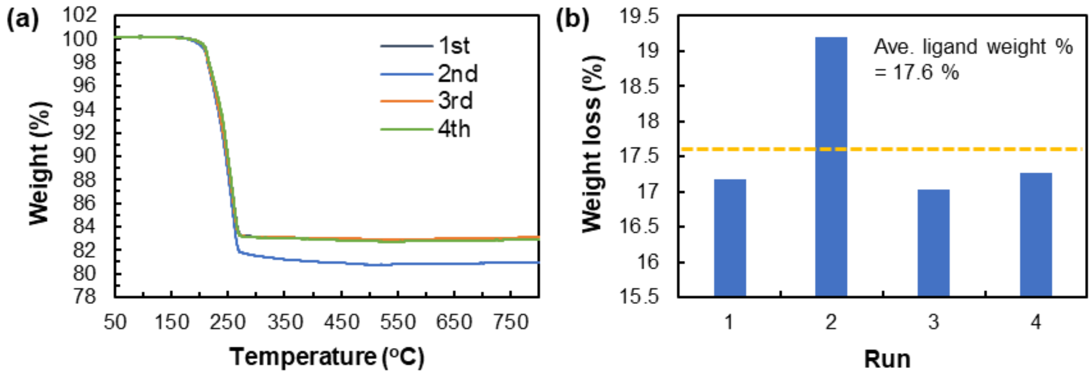

Figure S2. (a) TGA analysis of OT-AuNPs and (b) mass fraction of octanethiol ligands in AuNPs.

2. Gold ink formulation

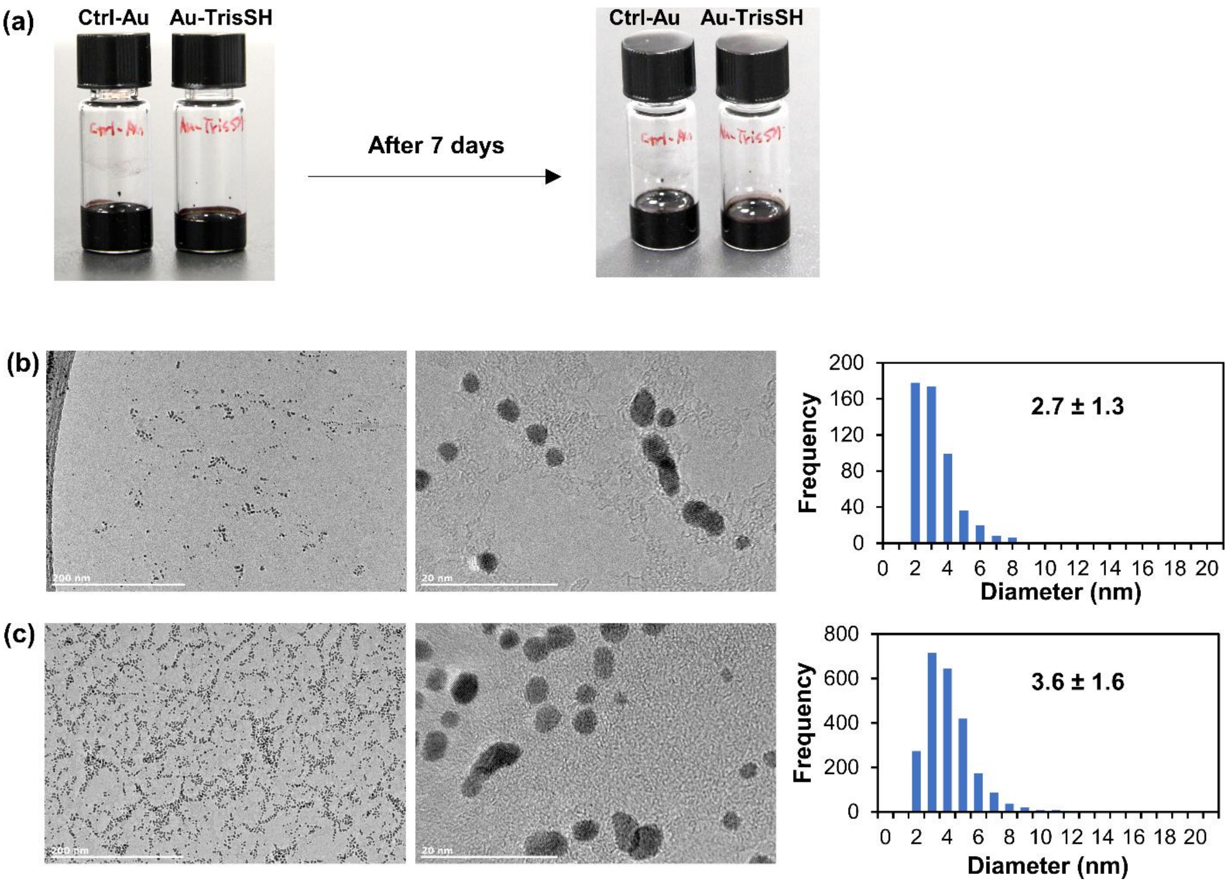

Figure S3. Stability of two gold ink formulations, Ctrl-Au and Au-TrisSH. (a) Photographs of (left) the dispersions of two gold inks with and without TrisSH as prepared and (right) the dispersions stored at ambient condition for 7 days. TEM images and histogram of size distribution of AuNPs from two dispersions, (b) Ctrl-Au ink and (c) Au-TrisSH ink, stored at ambient condition for 8 months.

3. Inkjet printing conductive gold inks

The inkjet printing resolution of Ctrl-Au and Au-TrisSH inks was studied on various substrates: borosilicate glass slide, silicon wafer, poly(ethylene terephthalate) (PET), and poly(ethylene naphthalate) (PEN) at room temperature and 90°C. The average single droplet diameters of both inks were measured using optical microscope (Figure S4).

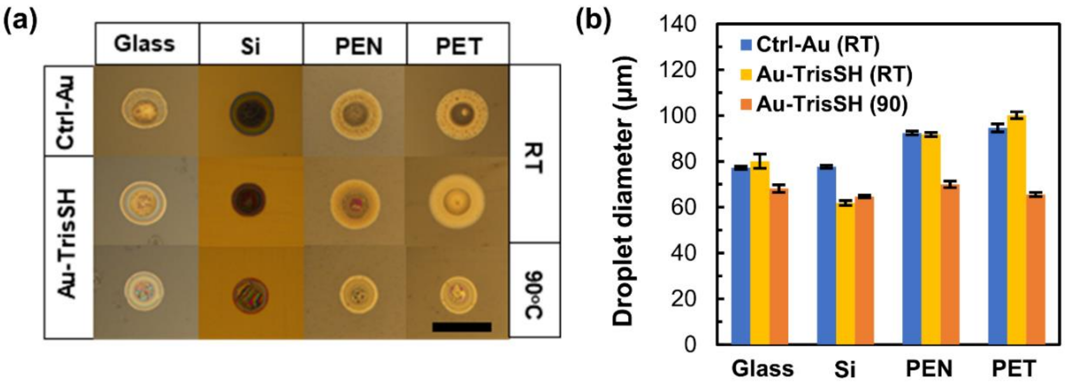

Figure S4. Printing resolution of Ctrl-Au and Au-TrisSH inks at different substrates and substrate temperatures. (a) Optical microscope images and (b) average diameters of single droplets of Ctrl-Au and Au-TrisSH inks at different substrates including glass slide, silicon wafer, PEN and PET and at different substrate temperatures of room temperature (RT) and 90°C.

3. Characterization of inkjet printed gold structures

For a single layer print, the layer thickness was found to be 163 ± 24 nm from FIB-SEM images of the cross section shown in Figure S5.

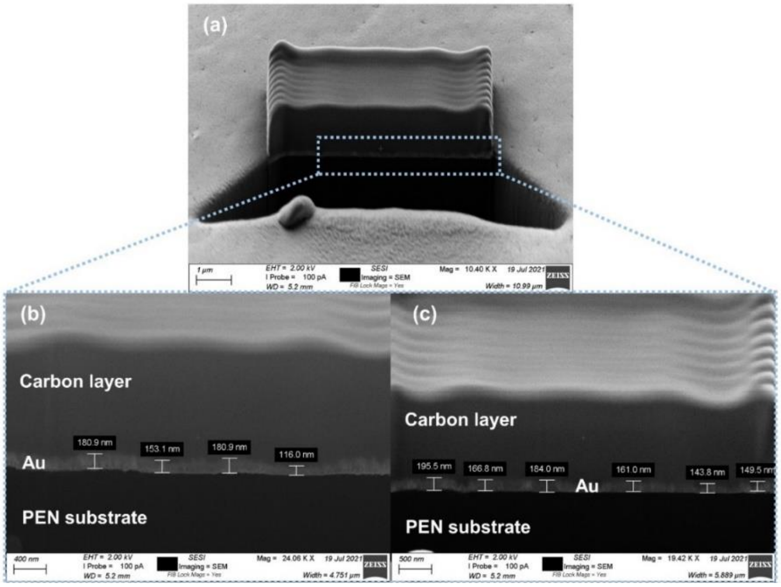

Figure S5. FIB-SEM images of the cross section of a single layer printed gold structure on a PEN substrate (Ctrl-Au) for thickness measurement with magnification of (a) 10.40K, (b) 24.06K and (c) 19.42K.

4. Electrical properties of inkjet printed gold structures

4.1. Thermal activation energy of the sintering process

Thermal activation energy[1, 2] was calculated from the following equation:

$$\sigma\,(n,T) = \sigma_o \exp\,(-n\beta_n) \exp\,(-\frac{E_A}{RT}) \quad \text{(S1)}$$

,where σ_o the intrinsic conductivity of nanoparticles, n is alkanethiolate chain length, β_n the corresponding electronic coupling term, E_A the activation energy of conductivity (kJ/mol), R the universal gas constant (8.3145 J/mol·K) and T the temperature in K.

(a)

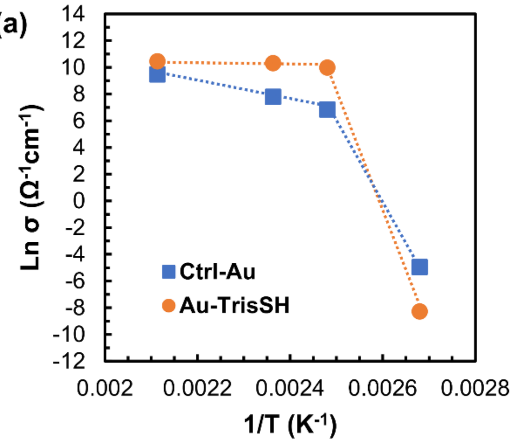

(b)

Temp range (°C)	E_A (KJ/mol)	
	Ctrl-Au	Au-TrisSH
100-130	7.11	11.01
130-200	0.85	0.14

Figure S6. (a) Arrhenius plot of electrical conductivities of single printed layers of Ctrl-Au and Au-TrisSH inks sintered at the temperature range from 100°C to 200°C and (b) activation energies calculated from Arrhenius plot.

4.2. Leak current test

In the structure of inkjet printed Au/SiO_2/Si structure, the gate is preserved up to the applied gate voltage of ±100 V under ambient conditions, proving no migration and diffusion of Au through the SiO_2 layer of 200 nm (Figure S7).

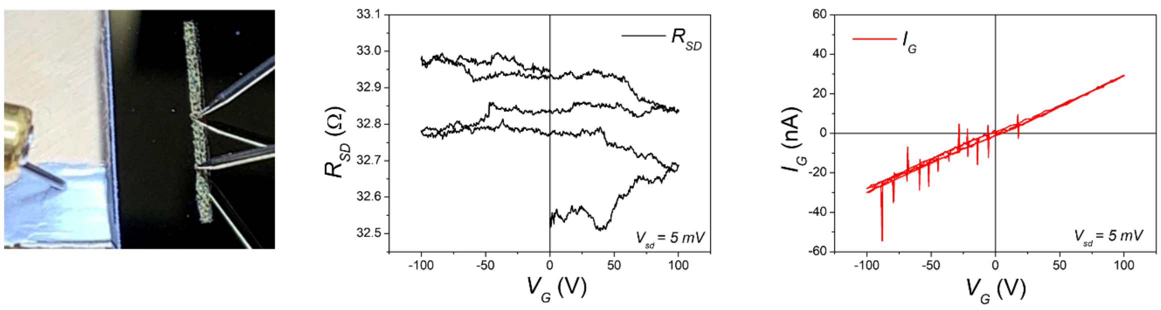

Figure S7. (a) A picture of the leakage current test of inkjet printed Au/SiO_2/Si structure, (b) the resistance (R_{SD}) while the gate voltage (V_G) is swept between -100 V and 100V, and (c) the gate leakage current (I_G) vs gate voltage (V_G).

4.3. Electrical performance stability

Cyclic bending deformation studies were performed on single printed layer electrodes (20 mm × 1 mm) on a PEN substrate sintered at 150°C. The electrical resistance was monitored through 1000 cycles of bending to the curvature $r \leq 0.6$ cm.

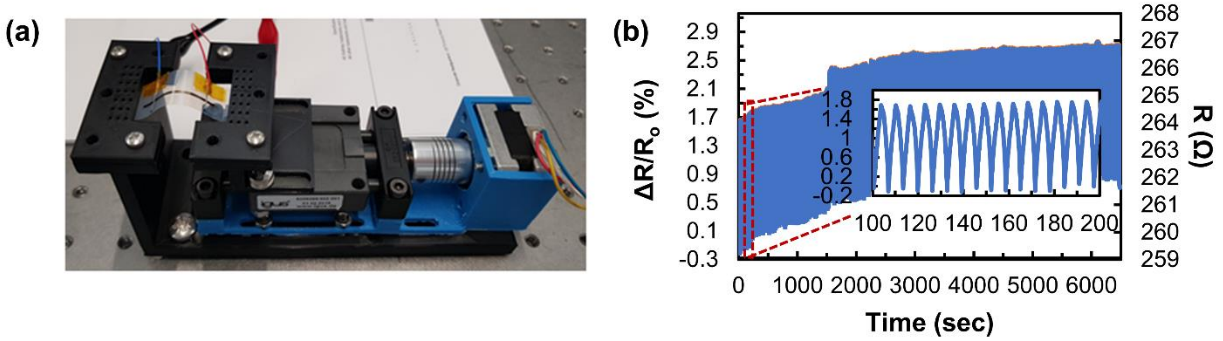

Figure S8. (a) A picture of bending test setup and (b) normalized electrical resistance change ($\Delta R/R_o$, %) vs time over 1000 bending cycles (bending radius (r) up to 0.6 cm) of Ctrl-Au. The inset graph shows the responses between 100 and 200 sec of testing time.

5. Adhesion between an inkjet printed Au film and a polymer substrate

The Scotch tape peel test was performed to estimate the adhesion of an inkjet printed Au film on a polymer substrate.

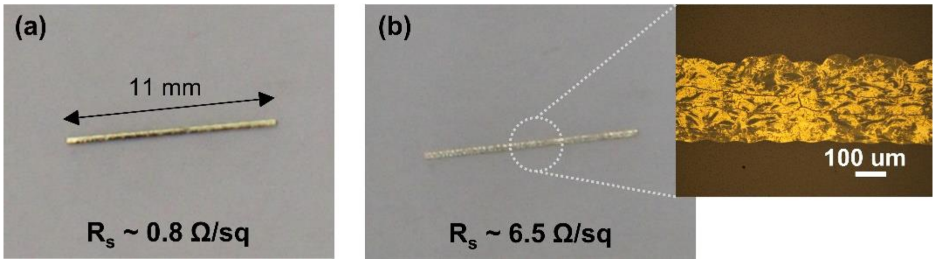

Figure S9. (a) A photograph of an inkjet printed Au-TrisSH layer (0.2 mm × 11 mm) before Scotch tape peel test. The sheet resistance (R_s) of the printed layer is 0.8 Ω/sq. (b) A photograph of an inkjet printed Au-TrisSH layer after Scotch tape peel test and (inset) its optical microscope image. R_s ~ 6.5 Ω/sq.

5. Chemical composition of inkjet printed gold structures

To confirm the role of TrisSH as cohesion enhancer, the surface chemical and molecular composition of printed structures was studied using Orbitrap secondary ion mass spectrometry (OrbiSIMS).

(a)

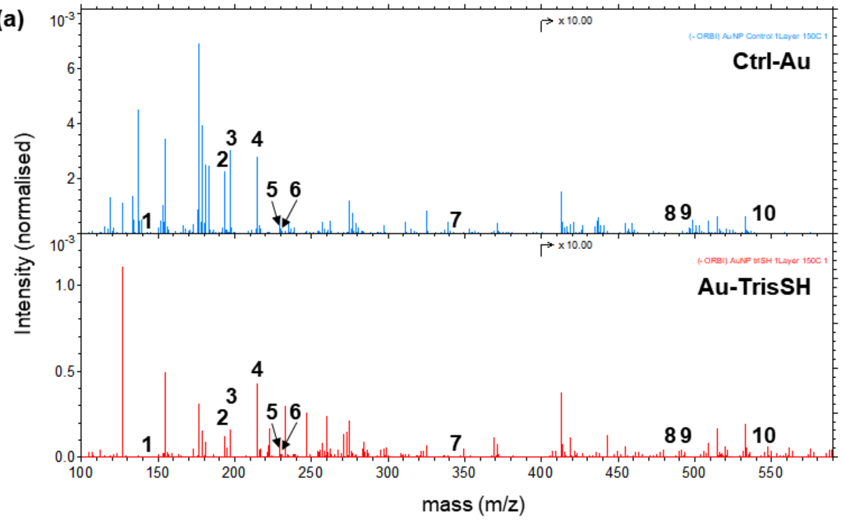

(b)

Label	Mass (m/z)	Formula
1	145.1056	$C_8H_{17}S^-$
2	193.0904	$C_8H_{17}SO_3^-$
3	196.9672	Au^-
4	213.9699	$AuOH^-$
5	228.9393	AuS^-
6	229.9471	$AuHS^-$
7	343.0801	$C_8H_{18}SAu^-$
8	487.1775	$C_{16}H_{34}S_2Au^-$
9	488.1808	$C_{16}H_{35}S_2Au^-$
10	539.0391	$C_8H_{17}SAu_2^-$

Figure S10. OrbiSIMS data from the surfaces of single layer printed gold structures of Ctrl-Au and Au-TrisSH on a silicon wafer substrate and sintered at 150ºC. (a) Mass spectra of the surfaces of the printed structures of Ctrl-Au and Au-TrisSH (b) peak assignment table. The intensity was normalized to the total ion counts.

REFERENCES

(1) Wuelfing, W. P.; Green, S. J.; Pietron, J. J.; Cliffel, D. E.; Murray, R. W., Electronic conductivity of solid-state, mixed-valent, monolayer-protected Au clusters. *J. Am. Chem. Soc.* **2000,** *122*, 11465-11472.

(2) Wang, G. R.; Wang, L.; Rendeng, Q.; Wang, J.; Luo, J.; Zhong, C.-J., Correlation between nanostructural parameters and conductivity properties for molecularly-mediated thin film assemblies of gold nanoparticles. *J. Mater. Chem.* **2007,** *17*, 457-462.
